# Enhanced salt-tolerance of *Bacillus subtilis* glutaminase by fusing self-assembling amphipathic peptides at its N-terminus

**DOI:** 10.3389/fbioe.2022.996138

**Published:** 2022-09-07

**Authors:** Song Liu, Shengqi Rao, Xiao Chen, Jianghua Li

**Affiliations:** ^1^ Science Center for Future Foods, Jiangnan University, Wuxi, Jiangsu, China; ^2^ National Engineering Laboratory for Cereal Fermentation Technology, Jiangnan University, Wuxi, Jiangsu, China; ^3^ School of Biotechnology, Jiangnan University, Wuxi, Jiangsu, China; ^4^ College of Food Science and Engineering, Yangzhou University, Yangzhou, Jiangsu, China

**Keywords:** glutaminase, self-assembling amphipathic peptides, temperature stability, salt tolerance, oligomerization

## Abstract

Glutaminase (EC 3.5.1.2) can catalyze the deamidation of glutamine, which has been used to improve umami taste in oriental fermented foods. However, a high salt concentration is still a fundamental challenge for glutaminase application, especially in soy sauce production. To improve the salt tolerance of glutaminase, the self-assembling amphiphilic peptides EAK16 and ELK16 were fused to the N-terminus of a mutant (E3C/E55F/D213T) derived from *Bacillus subtilis* glutaminase, yielding the fusion enzymes EAK16-E3C/E55F/D213T and ELK16-E3C/E55F/D213T, respectively. As ELK16-E3C/E55F/D213T was expressed as insoluble active inclusion bodies, only the purified EAK16-E3C/E55F/D213T was subjected to further analyses. After the incubation with 18% (w/v) NaCl for 200 min, the residual activities of EAK16-E3C/E55F/D213T in a NaCl-free solution reached 43.6%, while E3C/E55F/D213T was completely inactivated. When the enzyme reaction was conducted in the presence of 20% NaCl, the relative activity of EAK16-E3C/E55F/D213T was 0.47-fold higher than that of E3C/E55F/D213T. As protein surface hydrophobicity and protein particle size analysis suggested, oligomerization may play an important role in the salt-tolerance enhancement of the fusions. Furthermore, EAK16-E3C/E55F/D213T achieved a 0.88-fold increase in the titer of glutamic acid in a model system of soy sauce fermentation compared to E3C/E55F/D213T. Therefore, the fusion with self-assembling amphiphilic peptides is an efficient strategy to improve the salt-tolerance of glutaminase.

## Introduction

As a traditional condiment, soy sauce is famous in oriental countries because of its rosy color, special sauce, and umami taste enhancement. Among the flavor compounds in soy sauce, glutamic acid is the primary umami amino acid (Wakayama et al., 2005). The umami taste of soy sauce is safely and naturally enhanced by adding glutaminase (EC 3. 5. 1. 2), an enzyme that can produce glutamic acid from glutamine, which is plentiful in the fermentation broth (Binod et al., 2017). To be noted, a high salt concentration was required during soy sauce fermentation by *Aspergillus oryzae* Ye et al. (2013), and 15–18% (w/v) of sodium chloride is typically used (Koibuchi et al., 2000; Ito et al., 2013; Bazaraa et al., 2016). However, the catalytic activity of *A. oryzae* glutaminase was significantly inhibited at NaCl concentrations of over 15% (Masuo et al., 2005). In contrast to the salt-sensitive glutaminases, the enzymes resistant to salt are thus more favorable for soy sauce production.

To improve the umami taste of soy sauce, various salt-resistant glutaminases have been isolated in bacteria. The salt-resistant glutaminase, which showed a 30% increase in the catalytic activity at 16% NaCl, was firstly isolated from *Micrococcus luteus* K-3 (Moriguchi et al., 1993). The salt tolerance of *M. luteus* glutaminase was enhanced by removing its C-terminus *via* endogenous proteases (Yoshimune et al., 2004). In addition, researchers also reported the salt-resistant glutaminases from *Stenotrophomonas maltophilia* NYW-81 (Wakayama et al., 2005), *Bacillus* sp. LKG-01 (Kumar et al., 2012), *Bacillus amyloliquefaciens* (Ye et al., 2013), *Cohnella* sp. A01 (Mosallatpour et al., 2019), *Bizionia argentinensis* (Ferreira et al., 2021). Among them, the *Cohnella* glutaminase retained 90% of the catalytic activity in a 25% NaCl solution (Mosallatpour et al., 2019). Moreover, there are many reports on the salt-resistant glutaminases from fungi, such as *Cryptococcus nodaensis* (Sato et al., 1999), *A. oryzae* RIB40 (Masuo et al., 2005), and *Penicillium brevicompactum* NRC 829 (Elshafei et al., 2014). Statistical analyses have suggested that abundant glutamic acid residues on the glutaminase surface could explain their salt-tolerance mechanism (Yoshimune et al., 2006). To date, the salt-tolerant mechanism of glutaminase is still unclear, and there is no report on the molecular modification for enhanced salt tolerance of the enzyme.

Self-assembling amphipathic peptides (SAPs) are unique short peptides with alternating hydrophobic and hydrophilic residues, which can assemble into ordered nanostructures spontaneously (Yang et al., 2007). In our previous study, for the first time, the thermal stability of *Pseudomonas aeruginosa* lipoxygenase was improved by fusing SAPs to the enzyme N-terminus (Lu et al., 2013). To date, the fusion with SAPs has been successfully used to improve the thermal stabilities of different enzymes, such as polygalacturonate lyase (Zhao et al., 2018), L-asparaginase (Zhao et al., 2019), nitrile hydratase (Liu et al., 2014), and methyl parathion hydrolase (Shi et al., 2022). Compared to the wild-type enzyme, the enzymes fused with SAPs exhibited bigger particle sizes in solutions, suggesting that the oligomerization may account for the enzyme stabilization through the enhanced intermolecular hydrophobic interactions (Lu et al., 2013). The thermal stability of enzyme fusions could be further enhanced by adding NaCl, an accelerant of the intermolecular hydrophobic interactions (Zhao et al., 2019). Therefore, based on the stabilization mechanism, fusing SAPs is an efficient method to improve the salt-tolerance of glutaminase.

In this study, the salt tolerance of a mutant (E3C/E55F/D213T) derived from *B. subtilis* 168 glutaminase (GenBank No. 938416) was enhanced by fusing SAPs to its N-terminus *via* a PT linker. The mechanism for the enhanced salt tolerance was proposed based on protein surface hydrophobicity and particle size analysis. In addition, the glutamic acid generated by the enzyme fusions was examined using a modeled soy sauce fermentation system.

## Materials and methods

### Strains and plasmids

*Escherichia coli* JM109 was used as the host strain for gene cloning. For expressing E3C/E55F/D213T and its fusions, *Bacillus subtilis* WB600 and pP43NMK were used as the host and plasmid, respectively. The plasmid pP43NMK-E3C/E55F/D213T expressing the mutant E3C/E55F/D213T from *B. subtilis* 168 glutaminase was constructed in our previous study (Chen et al., 2019).

### Construction of the plasmids for expressing E3C/E55F/D213T fused with SAPs

**FIGURE 1:**
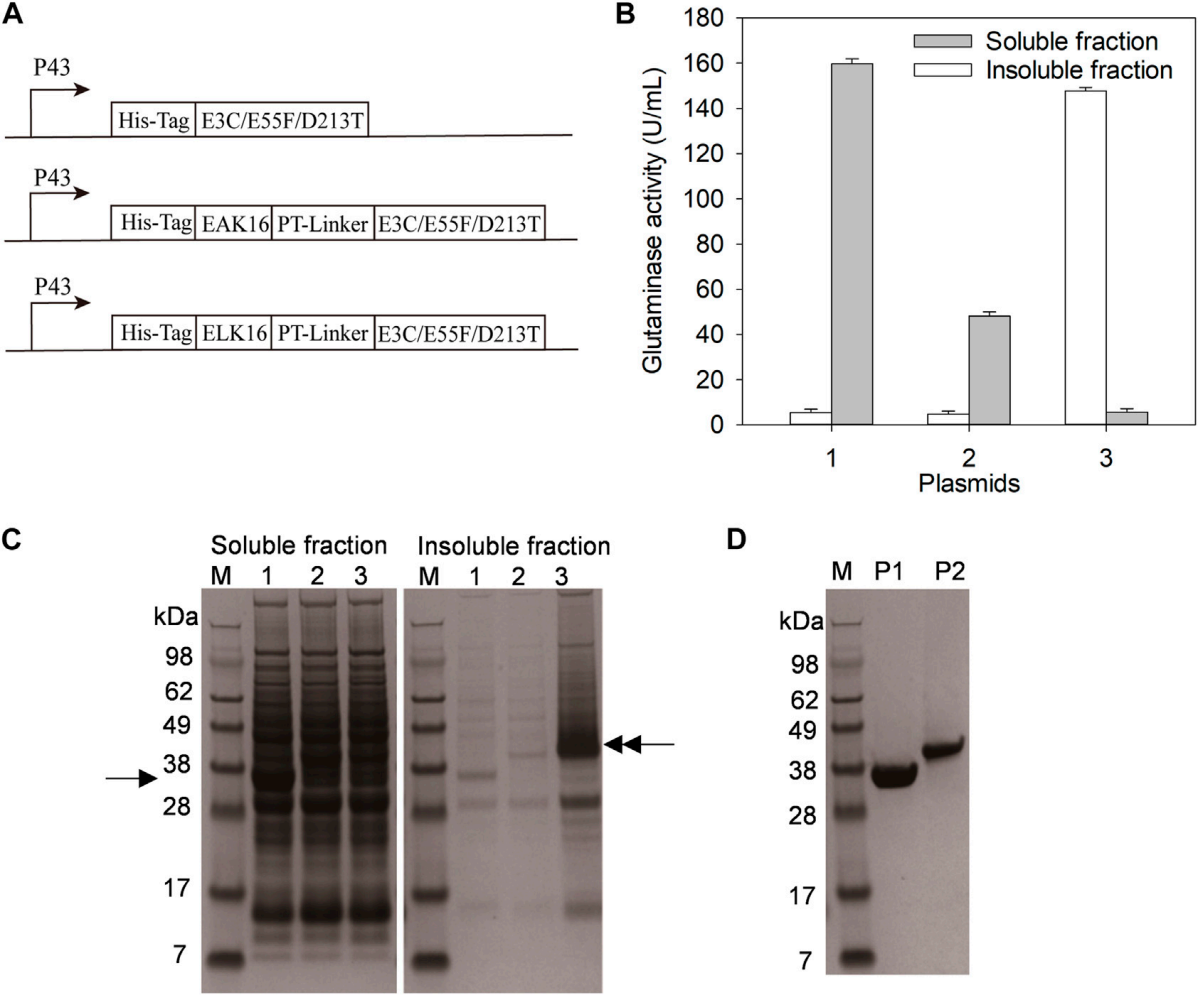
Expression and purification of E3C/E55F/D213T and its fusions. **(A)** The plasmids expressing E3C/E55F/D213T and its fusions. **(B)** Glutaminase determination. **(C)** SDS-PAGE analysis of enzyme expression. 1: *B. subtilis* WB600 carrying pP43NMK-E3C/E55F/D213T; 2: *B. subtilis* WB600 carrying pP43NMK-EAK16-E3C/E55F/D213T; 3: *B. subtilis* WB600 carrying pP43NMK-ELK16-E3C/E55F/D213T. **(D)** SDS-PAGE analysis of purified enzymes. P1: E3C/E55F/D213T; P2: EAK16-E3C/E55F/D213T.

The One-Step Cloning method was used to construct the plasmids for expressing E3C/E55F/D213T fused with SAPs. The gene fragments encoding E3C/E55F/D213T and pP43NMK were amplified from pP43NMK-E3C/E55F/D213T using the primer pair P43NMK-YbgJ-F/P43NMK-YbgJ-R (Table 1). The primer pair EAK16-F/EAK16-R was used to amplify the EAK16 with PT linker (Table 2) from pET22b (+)/S1-pgl (Zhao et al., 2019). Using ClonExpress II One Step Cloning Kit (Vazyme, Nanjing, China), both gene fragments were ligated to produce the plasmid pP43NMK-EAK16-E3C/E55F/D213T expressing E3C/E55F/D213T fused with EAK16 at N-terminus (Figure 1A). The pP43NMK-EAK16-E3C/E55F/D213T was then amplified by the primer pair ELK16-F/ELK16-R (Table 1) and ligated by the One-Sep Cloning method, yielding the plasmid pP43NMK-ELK16-E3C/E55F/D213T expressing E3C/E55F/D213T fused with ELK16 at N-terminus (Table 2 and Figure 1A).

**TABLE 1:** Primers used in this study.

Primer	Nucleotide Sequence (5′–3′)
pP43NMK-YbgJ-F	ACC​CCG​ACT​CCG​ACC​CCA​AAA​TGT​TTG​ATA​AAA​GAG​CAC​CAG​AAA​GAC​ATA​AAC​CCG
pP43NMK-YbgJ-R	TTT​CGC​TTC​TGC​TTC​TGC​GTG​GTG​GTG​GTG​GTG​GTG​CAT​GTG​TAC​ATT​CCT​CT
EAK16-F	GCA​GAA​GCA​GAA​GCG​AAA​GCC​AAA​G
EAK16-R	TGG​GGT​CGG​AGT​CGG​GGT​GGT​CGG
ELK16-F	GAA​ACT​CAA​ACT​AGA​ACT​AGA​ACT​GAA​ACT​CAA​ATG​GAT​ATC​GCC​TAC​TCC​G
ELK16-R	TCT​AGT​TTG​AGT​TTC​AGT​TCT​AGT​TCT​AGG​TGG​TGG​TGG​TGG​TGG​TGC​ATG​TGT

**TABLE 2:** SAPs and PT-linker used in this study.

Name	Amino Acid Sequence (Nucleotide Sequence)	Gravy Value
EAK16	AEAEAKAKAEAEAKAK (GCA​GAA​GCA​GAA​GCG​AAA​GCC​AAA​GCG​GAG​GCG​GAA​GCT​AAG​GCT​AAA)	−0.950
ELK16	LELELKLKLELELKLK(CTAGAACTAGAACTGAAACTCAAACTAGAACTAGAACTGAAACTCAAA)	0.050
PT-Linker	PTPPTTPTPPTTPTPTP(CCTACTCCGCCGACGACCCCGACCCCGCCGACCACCCCGACTCCGACCCCA)	−1.176

### Protein expression and purification

The plasmid pP43NMK-E3C/E55F/D213T, pP43NMK-EAK16-E3C/E55F/D213T, and pP43NMK-ELK16-E3C/E55F/D213T were transformed into *B. subtilis* WB600, yielding the strain expressing E3C/E55F/D213T, EAK16-E3C/E55F/D213T, and ELK16-E3C/E55F/D213T, respectively. The seed culture of each recombinant strain was prepared by growing cells in an LB medium containing 50 μg/ml kanamycin and cultured at 37°C for 8 h. Then, 2% seed culture was transferred into a TB medium with 50 μg/ml kanamycin and cultured at 37°C for 48 h.

After the fermentation broth was centrifuged at 7000 r/min for 15 min at 4°C, the cells were collected and resuspended in lysis buffer (50 mM K_2_HPO_4_-KH_2_PO_4_, pH7.5). Then, the cell suspensions were subjected to sonication for 30 min and centrifuged at 7000 r/min for 15 min at 4°C. The supernatants were subjected to affinity purification with His Trap^™^ FF (Qianchun Bio, Yancheng, China) after being filtered with a 0.22 μm filter membrane. The E3C/E55F/D213T and its fusions were eluted with elution buffer (50 mM imidazole, 20 mM K_2_HPO_4_-KH_2_PO_4_, pH 7.5) and desalted with K_2_HPO_4_-KH_2_PO_4_ buffer (20 mM, pH 7.5) using a Superdex G25 column (GE Healthcare, New Yorker, United States). The centrifugation sediments containing the active inclusion bodies of ELK16-E3C/E55F/D213T were washed with the lysis buffer twice and resuspended in the lysis buffer for further analysis.

### Catalytic property analyses

To test the salt tolerance, the purified E3C/E55F/D213T and its fusions were diluted to the same concentration in NaCl-phosphate buffer (0–18% (w/v) NaCl, 20 mM K_2_HPO_4_-KH_2_PO_4_, pH 7.5) and incubated at 50°C for 200 min. Samples were taken for glutaminase activity determination every 20 min, and the residual activity of each sample was obtained by dividing the sample activity by the initial activity before the salt stress. To test the effect of NaCl concentration on the catalytic reaction, the enzyme reactions were initiated by mixing 100 μL enzyme solution with the 1100 μL substrate solution (74 mM L-glutamine, 20 mM K_2_HPO_4_-KH_2_PO_4_, pH 7.5) containing 0–20% (w/v) NaCl. The residual activity at each NaCl concentration was obtained by dividing the enzyme activity at the NaCl concentration by the corresponding activity in the absence of NaCl.

The purified glutaminase mutants were diluted to the same concentration for the thermal stability determination. To obtain the half-life (*t*_1/2_), the glutaminase samples were incubated at a 55°C water bath and taken every 10 min. After cooled down on the ice, the enzyme solutions were subjected to glutaminase activity determination. The residual activity was presented as the activity of the sample after the heat treatment divided by its initial activity. The *t*_1/2_ of each enzyme sample was obtained using Origin 2019 exponential fit (OriginLab, Northampton, US).

### Construction of a model system of a soy sauce fermentation

According to the previous method described by Tomita et al. (1989), a model system of soy sauce fermentation (5 ml) was constructed with modifications. First, 0.5 g of soy protein isolate was dissolved in 5 ml Tris-HCl buffer (100 mM, pH 8.0), followed by incubating with 500 μL alkaline protease at 60°C for 2 h. Second, 500 μL compounded flavor enzyme (Angel Yeast, Yichang, China) was added and incubated at 50°C for 4 h. The enzyme reactions were terminated by the heat treatment at 90°C for 10 min. After the centrifugation at 12, 000 × g for 15 min at 4°C, the supernatant was tested for amino acids using the TNBS method (Fields, 1972) in order to confirm the hydrolysis degree of soybean protein. Third, NaCl was added to the supernatant at a final concentration of 18% (w/v), and the mixture was adjusted to pH5. At last, 2 U of purified EAK16-E3C/E55F or its fusions were added to the mixture at 45°C for 25 h. At certain intervals, a 200 μL of the reaction solution was taken for enzyme inactivation at 90°C for 10 min and subjected to L-glutamic acid determination.

### Protein analysis

SDS-PAGE analysis was performed on a vertical mini-gel apparatus (Bio-Rad, Hercules, United States) at 120 V for 1 h. The resolved protein bands were visualized by staining with Coomassie Brilliant Blue G-250 (Macklin, Shanghai, China) solution. Using bovine serum albumin as the standard, protein concentrations were measured using Detergent Compatible Bradford Protein Assay Kit (Beyotime, Shanghai, China).

### Glutaminase activity determination

Nessler^’^s reagent method was used to determine the glutaminase activity (Imada et al., 1973). The enzyme reaction was initiated by mixing 100 μL enzyme solution with the 1100 μL substrate solution (74 mM L-glutamine, 20 mM K_2_HPO_4_-KH_2_PO_4_, pH 7.5). After the incubation at 37°C for10 min, the reaction was terminated by adding 100 μL trichloroacetic acid solution (1.5 M), and the mixture was centrifuged at 12, 000 × g for 10 min. Then, 100 μL of the supernatant was added to 500 μL Nessler’s reagent diluted with 3400 μL deionized water, and the optical density was measured at a wavelength of 436 nm. One unit of glutaminase activity was defined as 1 μmol NH_3_ produced from L-glutamine per minute. Kinetic parameters (*K*_m_ and *k*_cat_) were determined using 10–100 mM L-glutamine solution.

### Determination of protein surface hydrophobicity

8-Anilino-1-naphthalene sulfonic acid (ANS) was used as the hydrophobic probe to detect protein surface hydrophobicity (Jasniewski et al., 2009). The purified enzymes were diluted to a final concentration of 10–100 μg/ml with phosphate buffer (20 mM K_2_HPO_4_-KH_2_PO_4_, pH 7.5), followed by adding ANS to a final concentration of 100 μM. After the incubation for 1 h in the dark, the mixture was subjected to fluorescence intensity analysis. The excitation and emission wavelengths are 390 and 470 nm, respectively. The surface hydrophobicity index is defined as the initial stage slope of the plot of fluorescence intensity versus the protein concentration.

### Dynamic light scattering analysis

The purified enzymes were diluted to 2 mg/ml in 0% and 15% (w/v) NaCl phosphate buffer (20 mM K_2_HPO_4_-KH_2_PO_4_, pH 7.5) and incubated at 25°C for 1 h. The enzyme solutions were subjected to the dynamic light scattering analysis at 25°C using Laser Nanometer Particle Size Analyzer (Malvern Instruments, Malvern, United Kingdom). The experimental data were analyzed using the built-in software (Dispersion Technology Software Version 4.10) to calculate the statistical value of the diameter size of the particles in each solution.

### Transmission electron microscopy analysis

A 10 μl of the purified enzymes was mixed with an equal volume of 3% (w/v) phosphotungstic acid for negative staining. Then, the mixture was incubated on the copper mesh, and the extra water was removed. Finally, the enzyme samples on the copper mesh were ready for transmission electron microscopy analysis on Hitachi H-7650B (Hitachi, Tokyo, Japan).

### L-glutamic acid determination

The content of L-glutamic acid was measured using the L-Glutamic acid Colorimetric-method Kit (R-Biopharm, Darmstadt, Germany).

## Results

### Preparation of the E3C/E55F/D213T fusions with EAK16 and ELK16

SAPs could vary in hydrophobicity due to their different amino acid compositions (Lu et al., 2013). In previous studies, the fusion with the less hydrophobic SAPs (e.g. EAK16) resulted in the formation of soluble homomultimeric enzymes (Lu et al., 2013), while that with the relatively strong hydrophobic SAPs (e.g. ELK16) generally produced active inclusion bodies (Wu et al., 2011). Based on the pP43NMK-E3C/E55F/D213T in which E3C/E55F/D213T was expressed under the P43 promoter, the plasmids pP43NMK-EAK16-E3C/E55F/D213T and pP43NMK-ELK16-E3C/E55F/D213T were constructed to fuse EAK16 and ELK16 to the N-terminus of E3C/E55F/D213T *via* a PT-linker, respectively (Figure 1A and Table 2). To facilitate affinity purification, all the constructs were fused with a His-tag upstream of E3C/E55F/D213T or SAPs (Figure 1A). The expression plasmids pP43NMK-E3C/E55F/D213T, pP43NMK-EAK16-E3C/E55F/D213T, and pP43NMK-ELK16-E3C/E55F/D213T were transformed into *B. subtilis* WB600, yielding the strains expressing E3C/E55F/D213T, EAK16-E3C/E55F/D213T, and ELK16-E3C/E55F/D213T, respectively. After fermentation in the TB medium, glutaminase activity determination and SDS-PAGE were used to examine both the soluble and insoluble fractions produced within the cells. As shown in Figure 1B, the vast majority of glutaminases were found in the soluble fractions of E3C/E55F/D213T and EAK16-E3C/E55F/D213T, whereas the enzyme activity was largely detected in the insoluble fraction of ELK16-E3C/E55F/D213T. SDS-PAGE analysis indicated that the band of E3C/E55F/D213T (40 kDa) in the soluble fraction was considerably more visible than those of E3C/E55F/D213T fusions (Figure 1C). In contrast, there was a thick ELK16-E3C/E55F/D213T fusion band (40 kDa) in the intracellular insoluble fraction (Figure 1C). Therefore, in *B. subtilis* WB600, E3C/E55F/D213T and EAK16-E3C/E55F/D213T likely to be expressed in soluble form, while ELK16-E3C/E55F/D213T mostly produced as active inclusion bodies. Ni^2+^ affinity chromatography was used to achieve homogeneity in the purification of E3C/E55F/D213T and EAK16-E3C/E55F/D213T for further characterization because the insoluble ELK16-E3C/E55F/D213T was not suitable for column purification (Figure 1D).

### The temperature stabilities of the E3C/E55F/D213T fusion in the presence of NaCl

**FIGURE 2:**
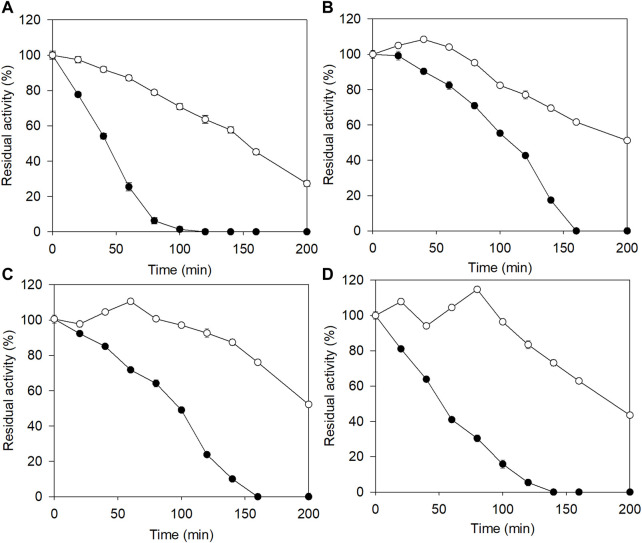
The effects of salt concentration on the stability of E3C/E55F/D213T and its fusion at 50 °C. **(A)** 0% (w/v) NaCl. **(B)** 10% (w/v) NaCl. **(C)** 15% (w/v) NaCl. **(D)** 18% (w/v) NaCl. ●: E3C/E55F/D213T; ○: EAK16-E3C/E55F/D213T.

The purified E3C/E55F/D213T and its fusion were incubated with 0–18% (w/v) NaCl at 50°C for 200 min to analyze their salt tolerance. As shown in Figure 2A, the residual activity of E3C/E55F/D213T rapidly decreased with the incubation time, and the enzyme was completely inactivated at 120–160 min under the selected NaCl concentrations. Notably, the enzyme inactivation rate of E3C/E55F/D213T was significantly reduced in the presence of 10% NaCl (Figure 2B), but further increases in NaCl concentration accelerate the inactivation (Figures 2C,D). Compared to E3C/E55F/D213T, EAK16-E3C/E55F/D213T also showed significant increases in salt tolerance when NaCl was added (Figure 2). Without NaCl, EAK16-E3C/E55F/D213T kept around 28% of the initial enzyme activity after 200 min of incubation (Figure 2A). EAK16-E3C/E55F/D213T had final residual activity of 51.2% and 52.2% at 10% and 15% NaCl, respectively (Figures 2B,C), and dropped to 43.6% at 18% NaCl (Figure 2D). Intriguingly, the residual activity of EAK16-E3C/E55F/D213T increased with the time in the early stages of the incubation with 10% NaCl (Figure 2B), which was not observed in the case of the treatment without NaCl (Figures 2C,D), suggesting the enzyme activation of the SAP fusion mediated by NaCl. The incubations with 15% and 18% NaCl made such enzyme activations more noticeable (Figures 2C,D). However, E3C/E55F/D213T did not show residual activity increases during the incubation in all cases.

### The catalytic activities of the E3C/E55F/D213T fusion in the presence of NaCl

**FIGURE 3:**
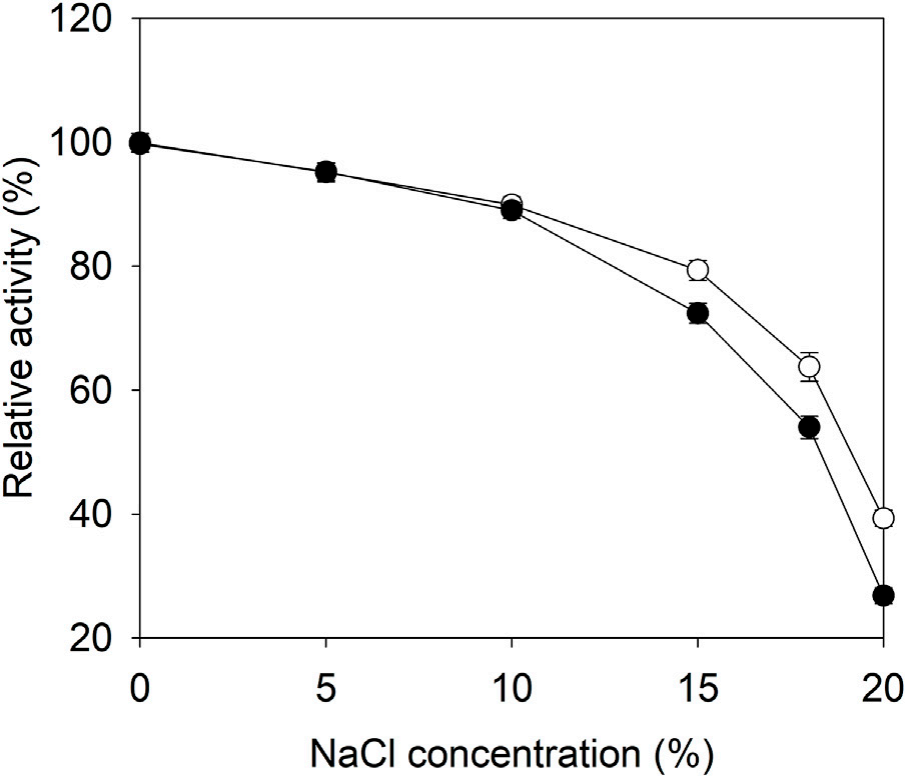
The effects of salt concentration on the catalytic activities of E3C/E55F/D213T and its fusion. ●: E3C/E55F/D213T; ○: EAK16-E3C/E55F/D213T.

To examine the effect of SAP fusion on the catalytic reaction, the glutaminase activity of E3C/E55F/D213T was determined at 37°C in the presence of 0–20% (w/v) NaCl. As shown in Figure 3, the relative activities of E3C/E55F/D213T and its fusion were decreased with the salt concentration, and they shared similar activities in the salt solution, in which the NaCl concentration was less than or equal to 10%. When the NaCl concentration was increased further, EAK16-E3C/E55F/D213T exhibited higher relative activities (Figure 3). Finally, the relative activities of EAK16-E3C/E55F/D213T in 20% NaCl solution were increased by 0.47-fold compared to E3C/E55F/D213T.

### The thermal stabilities and catalytic kinetics of the E3C/E55F/D213T fusion

The purified E3C/E55F/D213T and its fusion were subjected to thermal stability and enzyme reaction kinetics analysis. As shown in Table 3, the *t*_1/2_ value of EAK16-E3C/E55F/D213T at 55°C was 67% higher than that of E3C/E55F/D213T. EAK16-E3C/E55F/D213T revealed relatively lower *K*_m_ and higher *k*_cat_ values than E3C/E55F/D213T, resulting in an enhanced catalytic efficiency (*k*_cat_/*K*_m_).

**TABLE 3:** The thermal stability and kinetic parameters of mutants with SAP.

Enzymes	*t*_1/2_ (55°C) (min)	Specific Activity (U mg^−1^)	*K*_m_ (mM)	*K*_cat_ (10^3^ s^−1^)	*K*_cat_/*K*_m_ (s^−1^·mM^−1^)
E3C/E55F/D213T	29.47 ± 0.93	664 ± 6.3	22.72 ± 1.5	3.73 ± 0.19	162.23 ± 3.24
EAK16-E3C/E55F/D213T	49.17 ± 1.22	501 ± 5.8	18.45 ± 1.2	4.24 ± 0.12	173.84 ± 4.20

### Protein surface hydrophobicity and particle size of the E3C/E55F/D213T fusions

**FIGURE 4:**
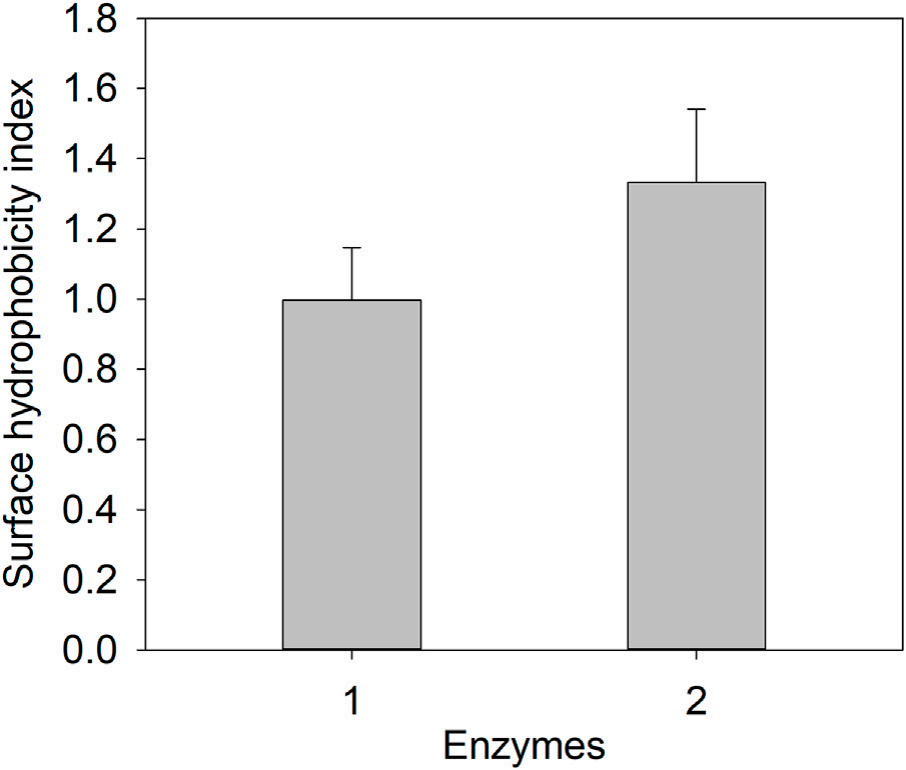
Protein surface hydrophobicity determination of E3C/E55F/D213T and its fusion. 1: E3C/E55F/D213T; 2: EAK16-E3C/E55F/D213T.

**FIGURE 5:**
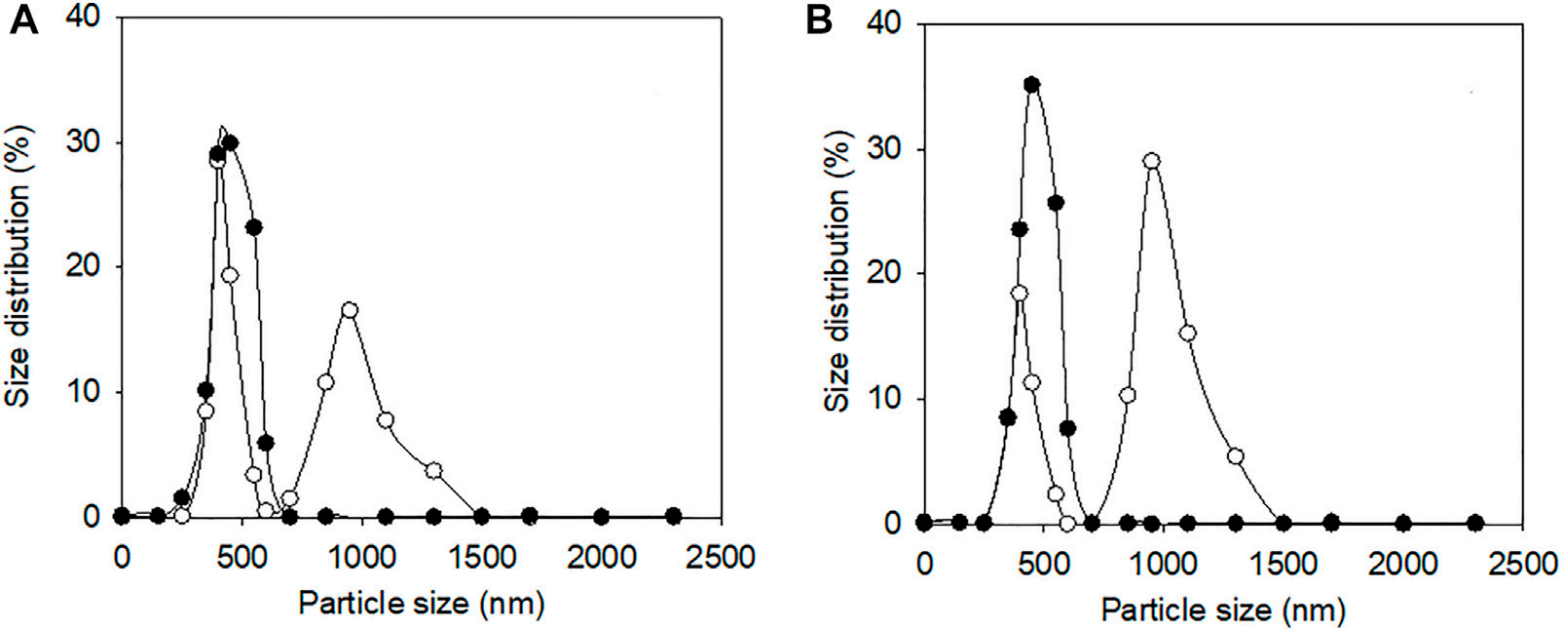
Dynamic light scattering analysis of E3C/E55F/D213T and its fusion in the presence of different NaCl Concentrations. **(A)** 0% (w/v) NaCl. **(B)** 15% (w/v) NaCl. ●: E3C/E55F/D213T; ○: EAK16-E3C/E55F/D213T.

**FIGURE 6:**
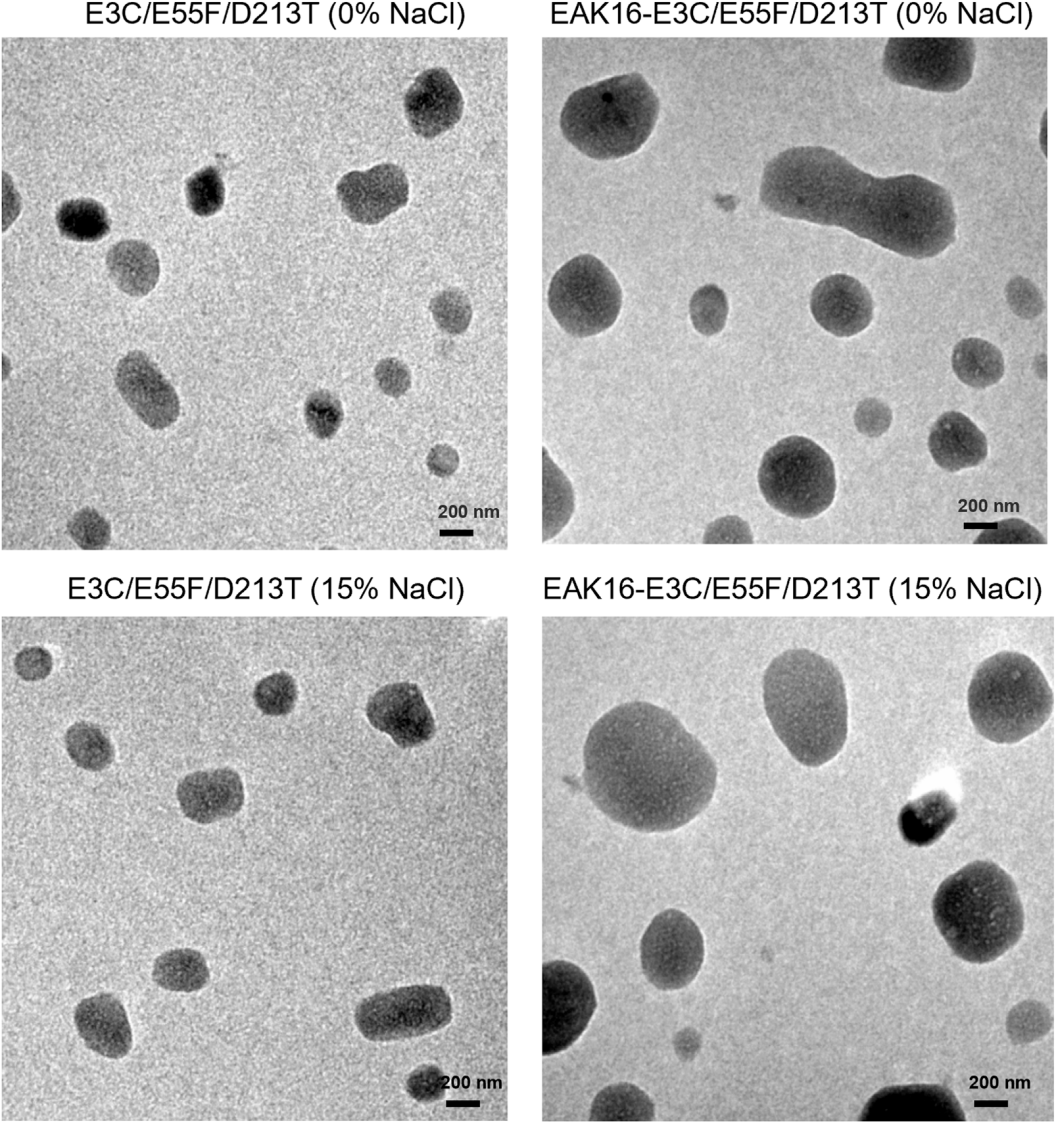
Transmission electron microscopy analysis of E3C/E55F/D213T and its fusion in the presence of different NaCl Concentrations.

Since ELK16-E3C/E55F/D213T was insoluble and not purified, only E3C/E55F/D213T and EAK16-E3C/E55F/D213T were tested for protein surface hydrophobicity using ANS as the hydrophobic probe. As seen in Figure 4, EAK16-E3C/E55F/D213T showed a 0.3-fold increase in the protein surface hydrophobicity index compared to E3C/E55F/D213T. The protein particle sizes of E3C/E55F/D213T and EAK16-E3C/E55F/D213T in 0% and 15% (w/v) NaCl solution were analyzed by dynamic light scattering. As shown in Figure 5, E3C/E55F/D213T displayed only one peak at 450 nm in both NaCl concentrations. Interestingly, EAK16-E3C/E55F/D213T had two peaks at 400 and 950 nm, respectively. Compared to the 15% NaCl solution, the NaCl-free solution generated a larger area for the peak at 400 nm and a smaller peak at 950 nm (Figure 5). The protein particle size in different NaCl solutions was also examined by transmission electron microscopy. For E3C/E55F/D213T, the protein particle size in the absence of NaCl was like that in 15% NaCl (Figure 6). In contrast to the solution without NaCl, the solution with 15% NaCl roughly reduced the proportion of small protein particles of EAK16-E3C/E55F/D213T (Figure 6). Compared to E3C/E55F/D213T, EAK16-E3C/E55F/D213T showed more big protein particles in both solution cases (Figure 6). These results suggested that the fusion with EAK16 increased the protein particle size of EAK16-E3C/E55F/D213T, which was increased at a high salt concentration.

### Glutamic acid production in the model system of soy sauce fermentation

**FIGURE 7:**
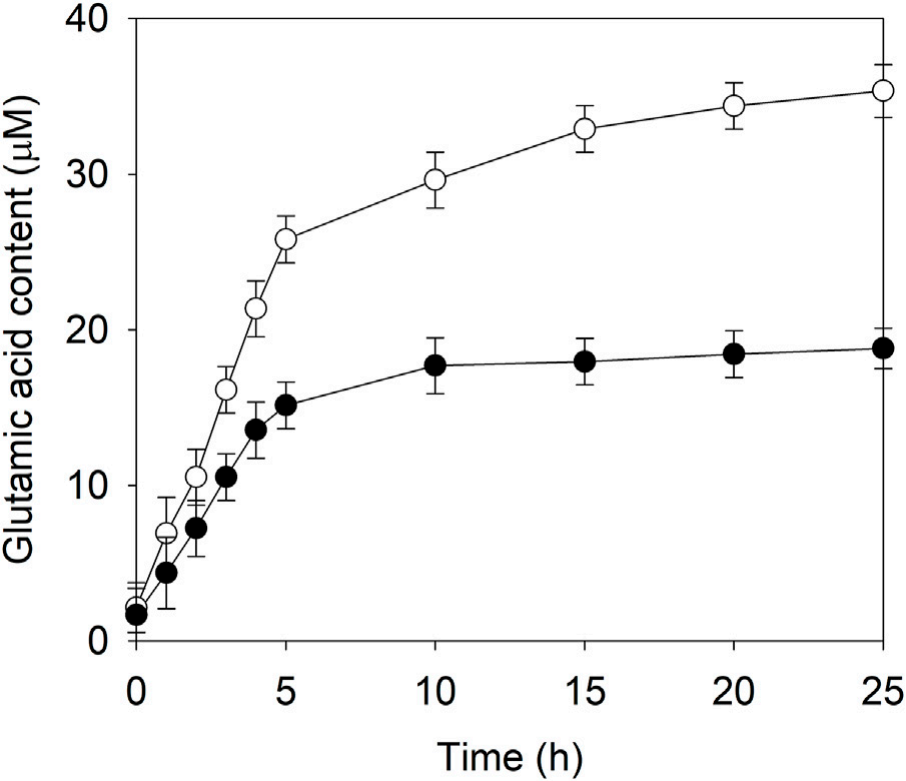
The glutamic acid content in the model system of soy sauce fermentation in the presence of E3C/E55F/D213T and its fusion. ●: E3C/E55F/D213T; ○: EAK16-E3C/E55F/D213T.

Soy protein isolate was sequentially digested by alkaline protease and compounded flavor enzyme in order to create a model system of soy sauce fermentation. After the enzyme inactivation and centrifugation, NaCl was added to the supernatant at a final concentration of 18% (w/v) and the pH was adjusted to 5. Then, EAK16-E3C/E55F/D213T or its fusion was introduced to improve the synthesis of glutamic acid at 45°C. During the initial 5 h reaction, the contents of glutamic acid in two cases rapidly increased with time, and EAK16-E3C/E55F/D213T showed a higher glutamic acid accumulation rate than E3C/E55F/D213T, (Figure 7). After that, the glutamic acid accumulation rates slowed down until the end of the reaction (Figure 7). Finally, the glutamic acid produced by EAK16-E3C/E55F/D213T was 0.88-fold higher than that done by E3C/E55F/D213T at 25 h.

## Discussion

Glutaminase catalyzes the production of L-glutamic acid from L-glutamine, which could improve the umami taste of soy sauce. Due to the high salt content (15–18% NaCl) required by soy sauce fermentation, many studies focused on the screening of salt-tolerant glutaminases from bacteria (Wakayama et al., 2005; Kumar et al., 2012; Mosallatpour et al., 2019; Ferreira et al., 2021) and fungi (Sato et al., 1999; Masuo et al., 2005; Elshafei et al., 2014). However, there are very few studies on the sat-tolerance enhancement of glutaminase *via* molecular modification. In the present study, the SAPs EAK16 (less hydrophobic) and ELK16 (relatively stronger hydrophobic) were fused to the N-terminus of the mutant E3C/E55F/D213T of *B. subtilis* glutaminase, yielding the fusions EAK16-E3C/E55F/D213T and ELK16-E3C/E55F/D213T, respectively. As ELK16-E3C/E55F/D213T was expressed as insoluble active inclusion bodies, only the purified EAK16-E3C/E55F/D213T was subjected to further analyses. As a result, the residual activity of EAK16-E3C/E55F/D213T reached 43.6% after the treatment with 18% NaCl solution for 200 min, while that of E3C/E55F/D213T was completely inactivated under the same treatment. Meantime, the relative activity of EAK16-E3C/E55F/D213T in the presence of 20% NaCl was 0.47-fold higher than that of E3C/E55F/D213T. In the case of 18% NaCl, the relative activity of EAK16-E3C/E55F/D213T reached 63.7%, suggesting that the salt tolerance of EAK16-E3C/E55F/D213T is higher than that of the glutaminase from *A. oryzae* RIB40 (Masuo et al., 2005)*.* As a result, EAK16-E3C/E55F/D213T achieved a 0.88-fold increase in the titer of glutamic acid in the model system of soy sauce fermentation, as compared to E3C/E55F/D213T. Therefore, the fusion with SAPs is an efficient strategy to improve the sat-tolerance of glutaminase.

In the absence of NaCl, EAK16-E3C/E55F/D213T exhibited higher temperature stability and catalytic activity than E3C/E55F/D213T. In particular, the surface hydrophobicity and the protein particle size of EAK16-E3C/E55F/D213T were higher than that of E3C/E55F/D213T. Similar results were also obtained in our previous study, in which EAK16 was fused to the N-terminus of lipoxygenase (Lu et al., 2013). Due to the relatively higher hydrophobicity of ELK16, ELK16-E3C/E55F/D213T was mainly expressed as active insoluble inclusion bodies, consistent with the results from *Bacillus subtilis* lipase A fused with the same SAP (Wu et al., 2011). Generally, hydrophobic interaction is one of the most important driving forces for protein oligomerization, which benefits their temperature stability (Vieille and Zeikus, 2001). In addition, it has been demonstrated that the enzymes in the packed state showed higher catalytic activity than those in the dispersed state due to increases in local substrate or cofactor concentration within the domain between enzyme surfaces, resulting from water entropic force (Dinh et al., 2020). Therefore, the hydrophobic interaction-mediated oligomerization or aggregation could play an essential role in the stabilization and activity enhancement of EAK16-E3C/E55F/D213T. A high concentration of salts is thought to lower enzyme solubility *via* a “salt-out effect” and is also expected to rigidify the enzyme’s hydrophobic core, decreasing the structural flexibility required for catalytic activity (Mevarech et al., 2000). This theory could well explain the enhanced temperature stability and reduced activity of E3C/E55F/D213T and its fusions in the presence of NaCl compared to the condition without NaCl. However, the residual activities of EAK16-E3C/E55F/D213T first raised and then fell during the incubation with 10–18% NaCl, which was not observed in the case of E3C/E55F/D213T. In addition, the incubation with NaCl increase protein particle size of EAK16-E3C/E55F/D213T, while E3C/E55F/D213T exhibited little changes. Therefore, NaCl improved the hydrophobic interaction-mediated oligomerization, resulting in the enhanced catalytic activity and stability. It has been demonstrated that natural salt-tolerant enzymes also tend to form oligomers *via* the special salt ion binding sites (Qiu et al., 2021). With enhanced temperature stability and activity in the presence of NaCl, both the enzyme fusions produce higher amounts of glutamic acid in the model system of soy sauce fermentation. Further investigation on SAP-mediated oligomerization of glutaminase should be done to understand the enhanced salt-tolerance, such as cryo-transmission electron microscopy under different salt concentrations and temperatures.

In addition to soy sauce fermentation, salt-tolerant enzymes are also favorable for fish sauce processing (e.g. subtilase) (Souza et al., 2012), and environmental remediation (e.g. tannase (Yao et al., 2011) and xylanase (Liu et al., 2013)). Recently, there has been a growing awareness that using freshwater resources has become a significant pressure for biorefineries. To be environmentally and economically sustainable, the replacement with seawater is a promising strategy in biorefineries (Scapini et al., 2022). In order to achieve such a replacement, a series of salt-tolerant enzymes are required for the enzymatic hydrolysis of lignocellulosic biomass, such as cellulases and xylanases [35]. However, there are few studies on protein engineering for enhanced salt tolerance. Thus, the strategy based on SAP fusion may also benefit the salt tolerance and application of the enzymes mentioned above.

## Data Availability

The datasets presented in this study can be found in online repositories. The names of the repository/repositories and accession number(s) can be found below: https://www.ncbi.nlm.nih.gov/genbank/, 938416.
